# A case of delayed diagnosis of disseminated coccidioidomycosis highlights opportunities for improved awareness

**DOI:** 10.1128/asmcr.00098-24

**Published:** 2025-04-11

**Authors:** Mylan Blomquist, Jacob Karsten, Brandon Larsen, Erin Graf

**Affiliations:** 1 Department of Laboratory Medicine and Pathology, Mayo Clinic, Phoenix, Arizona, USA; Vanderbilt University Medical Center, Nashville, Tennessee, USA

**Keywords:** coccidiomycosis, health equity, case report

## Abstract

**Background:**

As *Coccidioides* spp. are endemic in the southwestern United States, awareness of the risk factors for severe disease and dissemination is important to avoid delays in diagnosis and treatment. In addition to immunocompetent patients, individuals of certain ethnic backgrounds, especially African and Filipino ancestry, are at higher risk for dissemination. Diagnostic testing may be complicated by the lack of available invasive specimens for histopathologic examination and culture.

**Case Summary:**

A 29-year-old African American woman presented with a 6-year history of abdominal bloating and discomfort, which was previously diagnosed as endometriosis and possible irritable bowel syndrome. After multiple visits to primary care, urgent care, and emergency settings, she was referred for outpatient gynecology and gastrointestinal follow-up. Imaging revealed diffuse peritoneal nodules potentially concerning for malignancy, and biopsies revealed *Coccidioides* spp. spherules on the frozen section. Routine bacterial and fungal cultures from the biopsied nodules grew *Coccidioides immitis/posadasii*. The patient was placed on fluconazole with rapid symptom resolution and was maintained on fluconazole at a 2-year follow-up.

**Conclusion:**

The case presented highlights a reasonably common occurrence in endemic regions—the delayed diagnosis of coccidioidomycosis due to the lack of consideration as the etiology of the patient’s symptoms. This patient was at risk for dissemination due to her ethnicity, and while the differential for her presenting symptoms was broad, coccidioidomycosis should have been considered. In immunocompetent patients, like the case presented, serologic testing should be pursued, as severe or disseminated cases typically have positive IgG responses.

## INTRODUCTION

*Coccidioides immitis* and *Coccidioides posadasii* are soil-dwelling fungi endemic to the desert regions of the southwestern United States, northern Mexico, and regions of South America, with an expanding geographic range (1). Exposure to the fungal spores via inhalation can lead to pulmonary infection in both animals and humans. Most pulmonary infections result in asymptomatic clearance. A subset of individuals, estimated to be less than 30% of those exposed, will develop symptomatic pulmonary infections. Roughly 20–25% of community-acquired pneumonia in endemic regions is thought to be due to *Coccidioides* spp. (2, 3). Mild cases typically do not require antifungal therapy, while those with moderate to severe symptoms generally require prolonged treatment (4). Less than 1% of individuals with primary pulmonary infections develop disseminated infections; however, individuals with certain risk factors, including specific ethnic backgrounds, are at higher risk (5). Disseminated infections are associated with high morbidity and mortality, and diagnostic delays are common due to the lack of consideration of coccidioidomycosis as the etiology, even in endemic regions.

## CASE PRESENTATION

A 29-year-old G0P0 African American woman presented to an outside emergency department (ED) with bloating, abdominal cramping, diarrhea, early satiety, anorexia, an unintentional 10-lb weight loss, cough, dyspnea, chills, fever, and night sweats progressive over the course of a month. Her past medical history was significant for right total hip replacement 10 years prior for unspecified arthritis and chronic abdominal pain attributed to stage IV endometriosis and irritable bowel syndrome, both diagnosed 6 years prior. She had no family history of inflammatory bowel disease, gynecologic disease, or malignancy. She was a year-round Arizona resident. She had multiple outpatient visits for her abdominal symptoms over the course of the prior 6 years without resolution or definitive diagnosis. Upon presentation to the ED, she was treated with unspecified antibiotics without symptomatic relief and referred for outpatient evaluation by gynecology and gastroenterology at our institution.

Computed tomography (CT) scan of her abdomen and pelvis completed at her initial presentation to the ED revealed free fluid in the abdomen, thickening in the sigmoid colon, a 3-cm ovarian cyst, a pleural effusion, and a right upper lobe pulmonary nodule. Comprehensive laboratory testing performed by gastroenterology indicated normal liver function tests and normal stool studies. A complete blood count with differential was normal except for a slightly elevated eosinophil count of 0.49 cells per microliter (reference range 0.03–0.48). A colonoscopy confirmed the presence of thickening in the sigmoid colon, and biopsies showed nonspecific chronic inflammation of uncertain clinical significance, with no other evidence of colitis, active (acute) inflammation, or granulomas.

**Fig 1:**
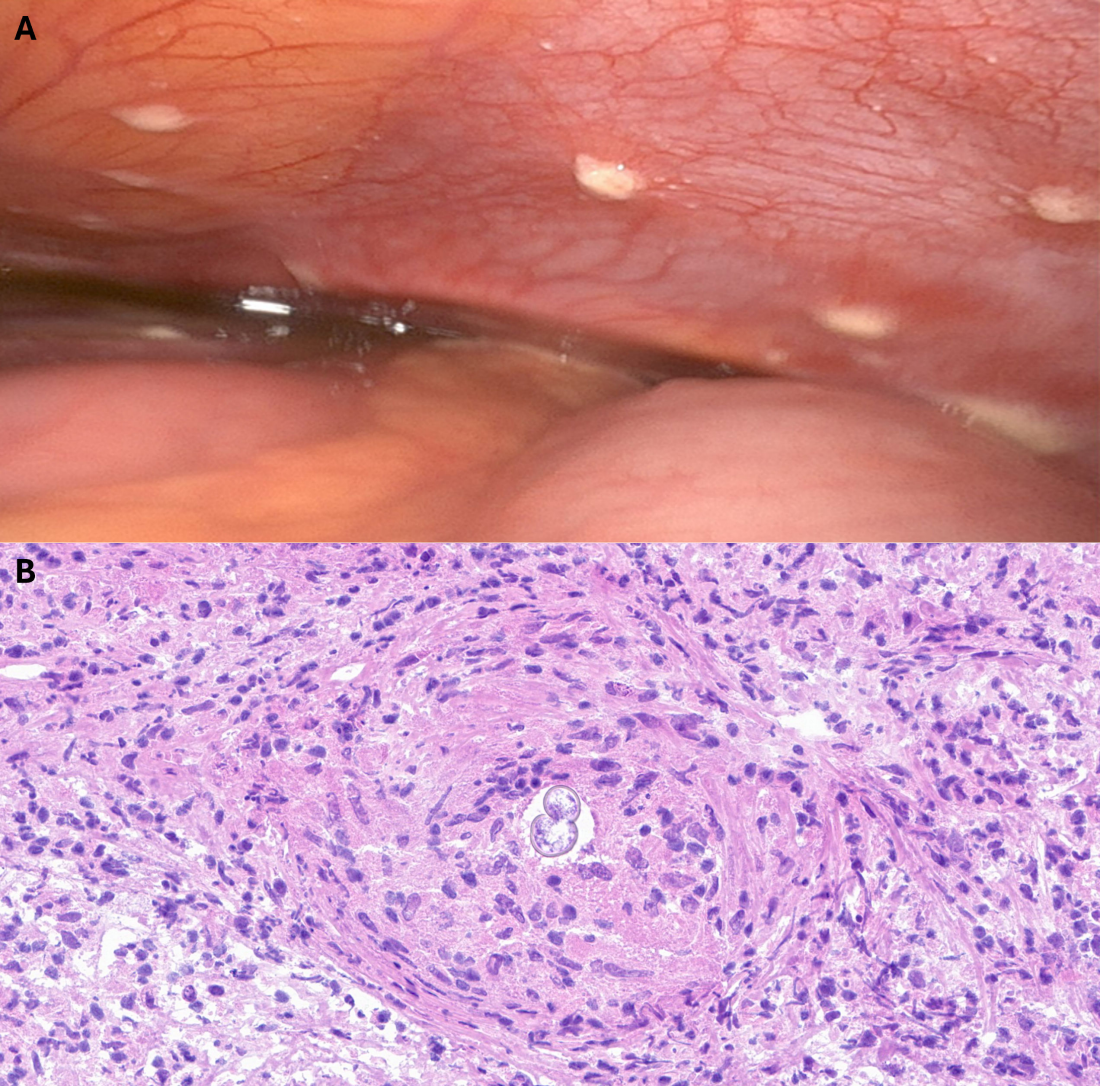
Case images. (**A**) Intraoperative photographs of peritoneal nodules and ascitic peritoneal fluid; (**B**).frozen section biopsy of a peritoneal nodule demonstrating spherules consistent with *Coccidioides* spp.

**Fig 2:**
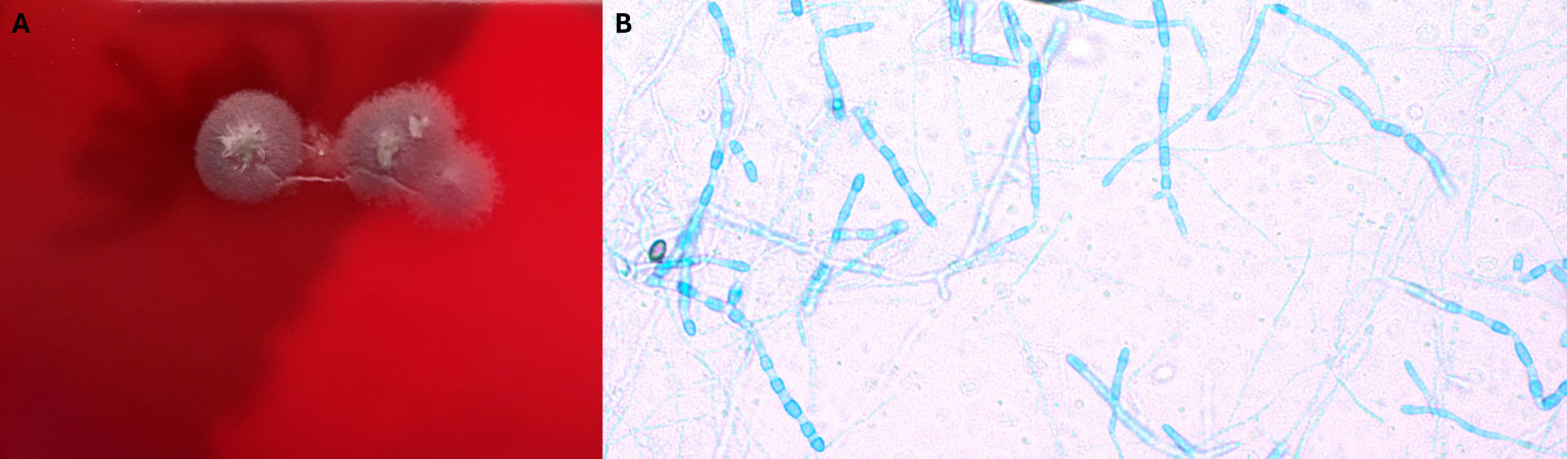
Culture findings. (**A**) Growth on sheep blood agar showing grey, glabrous colonies with characteristic “alpha hemolytic” type appearance consistent with *Coccidioides* spp.; (**B**) lactophenol aniline blue stain of a tape preparation demonstrating alternating arthroconidia characteristic of *Coccidioides* spp.

Magnetic resonance imaging of her pelvis completed by gynecology revealed findings suggesting deep pelvic endometriosis, extending into the uterosacral ligaments, large volume ascites with diffuse nodular thickening of the peritoneum, bilateral thickening and enhancement of the fallopian tubes, and bilateral ovarian cysts. Cancer Antigen 125 was elevated at 107 U/mL (reference range <46). Paracentesis was performed with a fluid cell count of 3,942 cells per uL (reference range <500), with 74% lymphocytes and 3% eosinophils (no reference range). Cytology showed chronic inflammation and no evidence of malignancy. Due to ongoing concern for malignancy, laparoscopy was performed, and nodules were noted throughout the entire peritoneal cavity (Fig. 1A) including the diaphragm, liver capsule, mesentery, uterus, fallopian tubes, and ovaries. Peritoneal biopsies were obtained for intraoperative consultation, and frozen section evaluation of the nodules revealed fungal spherules consistent with *Coccidioides* spp. (Fig. 1B). There was no histopathologic nor surgical evidence of endometriosis or malignancy. Three pelvic biopsy specimens were submitted for microbiologic cultures, two of which did not have fungal cultures ordered. All three grew mold consistent with *Coccidioides* spp., which were first identified on the sheep blood and chocolate agars of the bacterial cultures after 48 hours of incubation at 37°C and 5% CO_2_ (Fig. 2A). Tape preparations from colonies revealed characteristic alternating arthroconidia (Fig. 2B) and were later confirmed via laboratory-developed molecular testing to be *Coccidioides posadasii/immitis* (6). Minimum inhibitory concentration (MIC) testing is sent to a reference laboratory which demonstrates the MICs listed in Table 1. Antibody testing demonstrated positive IgM and IgG enzyme immunoassay as well as immunodiffusion reactions to *Coccidioides* spp. antigens and a complement fixation titer of 1:256 to *Coccidioides* spp. antigens. Urine antigen testing, performed at a reference laboratory, was negative. Fungal and bacterial cultures from the peritoneal fluid were negative.

**TABLE 1:** Antifungal MIC testing results

Antifungal agent	Minimum inhibitory concentration in mcg/mL
Fluconazole	4
Itraconazole	0.125
Posaconazole	0.06
Voriconazole	0.125
Isavuconazole	0.5

The patient was initiated on 400 mg of fluconazole therapy daily, with the dose increased to 800 mg daily shortly after. This latter dose was maintained for several months but was titrated back down to 400 mg due to hair loss, dry skin, and blurry vision.

Her abdominal distention and all other symptoms rapidly resolved shortly after starting fluconazole. Approximately 2 years after initiation of fluconazole therapy, her complement fixation titer decreased to 1:4, and she has remained free of abdominopelvic distention and cramping.

## DISCUSSION

The case presented highlights a common occurrence in endemic regions—the delayed diagnosis of coccidioidomycosis due to the lack of consideration as the etiology of the patient’s symptoms (7). In this case, the cause for delayed diagnosis was likely the nonspecific symptoms attributed to more common etiologies such as endometriosis, irritable bowel syndrome, or malignancy. It is unclear how far back in the patient’s initial course of symptoms *Coccidioides* spp. was the true culprit; however, both the infectious disease physician and gynecologist suspected that the original diagnosis of endometriosis 6 years prior was likely inaccurate and coccidioidomycosis could have been the likely cause all along. In a study within an Arizona hospital system, Ginn et al. found that 46% of patients had delays in the diagnosis of coccidioidomycosis of greater than 1 month, concomitant with increases in billed health care charges (8). Another study in Arizona found that only 13%, or fewer, of patients presenting with community-acquired pneumonia (CAP) were tested for *Coccidioides* spp. antibodies, despite roughly 25% of all community-acquired pneumonia being attributed to *Coccidioides* spp. in endemic regions (3). The reasons for this lack of consideration of coccidioidomycosis as a part of the differential are not entirely clear. A study conducted by colleagues at our institution found that emergency department providers were unlikely to order *Coccidioides* spp. testing on patients presenting with CAP primarily due to the difficulty in arranging follow-up, as no rapid testing options exist (2). After an educational intervention, serologic testing orders temporarily increased but returned to baseline after several months. These studies, and the case presented, highlight significant gaps in clinical practice, as delays in diagnosis increase the risk of morbidity and mortality, particularly for disseminated infections.

There are well-established risk factors for disseminated coccidioidomycosis, including immunocompromising conditions, and individuals from endemic regions should be screened for *Coccidioides* spp. antibodies prior to the start of immunosuppressive regimens and offered prophylaxis. Individuals from certain ethnic populations, including those without any known immunocompromising conditions, are at increased risk of dissemination, including individuals with African or Filipino ancestry, with a higher incidence in male sex assigned at birth. While the basis of this increased risk is not fully understood, research has shown certain genetic mutations associated with poor β-glucan sensing, and responses are enriched in individuals with disseminated coccidioidomycosis (9). There is recent evidence to suggest individuals with African or Filipino ancestry should be treated for mild disease due to the high degree of recurrence and dissemination in this population (4, 7).

*Coccidioides* spp. can disseminate to and infect any organ. The most common extrapulmonary sites of dissemination include skin and soft tissues, bone (particularly the spine), and the central nervous system. Involvement of the peritoneal cavity and/or genitourinary tract, like the case presented, is rare. A review of peritoneal coccidioidomycosis cases from academic medical centers in endemic regions of California only identified 17 cases over a 10-year period (2009–2019) (10). Patients were relatively young (mean age 44 years); 7 of 17 patients were of African American ethnicity, and the majority presented with non-specific abdominal pain and swelling—features similar to the case presented. Treatment of disseminated disease is typically, at minimum, several years of fluconazole, or other azole, followed by careful monitoring. Intravenous lipid amphotericin may be used initially for moderate to severe disease when more than one organ system is involved. Relapse of dissemination is extremely common, particularly for central nervous system infections, which require lifelong antifungal therapy due to the static activity of azoles. Antifungal agents can have unpleasant side effects, like the case presented, and may require dosage modifications for longer-term usage.

Laboratory testing for *Coccidioides* spp. infections is often a multistep approach. Culture and histopathology continue to be the gold standards to prove disease; however, both may lack sensitivity depending on sampling technique. *Coccidioides* spp. will grow readily on routine nonselective bacterial culture media (i.e., sheep blood and chocolate agars) as well as routinely used fungal culture media. This is important for laboratorians to recognize since cultures should only be manipulated in a biosafety cabinet preferably under BSL-3 conditions, due to the high risk of occupational exposure to arthroconidia spores (11). While *Coccidioides* spp. frequently grows from a variety of sources within 2 to 3 days on routine cultures, fungal cultures with extended incubation are always recommended, as cases may have delayed growth or competition with commensal flora from non-sterile sources. There are no established clinical breakpoints for antifungal agents and *in vitro* minimum inhibitory concentrations, though MICs of 16 ug/mL or higher to fluconazole are considered elevated, and elevated MICs to mold-active azoles are rare (12, 13). Molecular and antigen testing, only available at select reference laboratories, may have a role in improving the sensitivity of diagnosis of disseminated coccidioidomycosis, particularly in central nervous system infections (6, 14). Importantly, the case presented had a negative urine antigen test for *Coccidioides* spp. despite the overwhelming burden of infection in her peritoneal cavity, suggesting limitations of this testing approach to rule out disseminated disease. Peripheral, CSF, and other sterile fluid eosinophilia are common associations with coccidioidomycosis, as well as other fungal infections, but lack sensitivity and specificity as a diagnostic. Antibody testing remains a standard of care approach, especially when the infected site cannot be sampled (15). Three laboratory methods are routinely used for antibody testing, with recent guidance suggesting a cascade approach to use (4). Enzyme immunoassay (EIA) testing is now recommended as the frontline test and the most sensitive method. Unlike many infectious processes, IgG antibodies to *Coccidioides* spp. correlate with active infection, such that IgG reactivity disappears, albeit slowly in disseminated cases, as symptoms resolve. As a result, a positive *Coccidioides* spp. IgG is highly specific in an appropriately symptomatic individual. IgM is frequently co-positive with IgG in active disease, though not always, while a standalone positive IgM can be less specific. Antibody responses can be delayed during acute disease; thus, repeat EIA testing is recommended. However, in disseminated disease, which is a later-stage manifestation, IgG antibodies to *Coccidioides* spp. should be detectable in immunocompetent patients with high sensitivity and specificity. In order to avoid delays in diagnosis, EIA testing would be the recommended initial testing approach for patients who may be at risk for disseminated disease with potentially compatible symptoms, like the case presented. EIA testing is not cost-prohibitive for broad use in patients with compatible symptoms in or with exposure to endemic areas, with a Medicare reimbursement rate of $11.47 as of quarter 1 of 2025 (16). Immunocompromised patients may have blunted or no immune responses on any of the serologic testing methods. In such cases, culture and other non-antibody testing mentioned above should be aggressively pursued if clinical suspicion is high.
